# Long-Range Dependence Involutional Network for Logo Detection

**DOI:** 10.3390/e25010174

**Published:** 2023-01-15

**Authors:** Xingzhuo Li, Sujuan Hou, Baisong Zhang, Jing Wang, Weikuan Jia, Yuanjie Zheng

**Affiliations:** School of Information Science and Engineering, Shandong Normal University, Jinan 250358, China

**Keywords:** object detection, logo detection, feature fusion, attention mechanism

## Abstract

Logo detection is one of the crucial branches in computer vision due to various real-world applications, such as automatic logo detection and recognition, intelligent transportation, and trademark infringement detection. Compared with traditional handcrafted-feature-based methods, deep learning-based convolutional neural networks (CNNs) can learn both low-level and high-level image features. Recent decades have witnessed the great feature representation capabilities of deep CNNs and their variants, which have been very good at discovering intricate structures in high-dimensional data and are thereby applicable to many domains including logo detection. However, logo detection remains challenging, as existing detection methods cannot solve well the problems of a multiscale and large aspect ratios. In this paper, we tackle these challenges by developing a novel long-range dependence involutional network (LDI-Net). Specifically, we designed a strategy that combines a new operator and a self-attention mechanism via rethinking the intrinsic principle of convolution called long-range dependence involution (LD involution) to alleviate the detection difficulties caused by large aspect ratios. We also introduce a multilevel representation neural architecture search (MRNAS) to detect multiscale logo objects by constructing a novel multipath topology. In addition, we implemented an adaptive RoI pooling module (ARM) to improve detection efficiency by addressing the problem of logo deformation. Comprehensive experiments on four benchmark logo datasets demonstrate the effectiveness and efficiency of the proposed approach.

## 1. Introduction

Feature extraction is the most fundamental problem in various image-related tasks. Recent years have witnessed the powerful feature representation capability of convolutional neural networks (CNNs) that makes them very good at extracting rich image features. This is because they have two inherent advantages, namely, sparse connectivity and weight sharing. The former changes the situation of full connectivity in traditional neural networks and enables local perception, while the latter allows for the number of parameters to be significantly reduced, thus allowing for CNNs to train light models with fewer parameters. These advantages enable CNNs to outperform the traditional manual feature method. Therefore, deep-learning-based CNNs are increasingly dominant in object detection tasks. As a special form of object detection, logo detection aims at finding all the logos in an image or a video and return their locations. Extracting effective features is a crucial step in logo detection, in which deep CNNs can help in discovering intricate structures in logo datasets.

Logo detection plays an important role in various real-world applications, such as intelligent transportation [1,2], trademark infringement detection [3], and automatic logo detection and recognition [4]. However, logo detection is a complex task compared to general object detection because logo images usually have two distinctive characteristics: a large aspect ratio and multiple scales. On the one hand, there are many long words, artistic words, and feature images in a logo image resulting in a relatively large aspect ratio. On the other hand, the exquisite design of logo images causes a variety of multiscale logo objects in an image.

**Figure 1:**
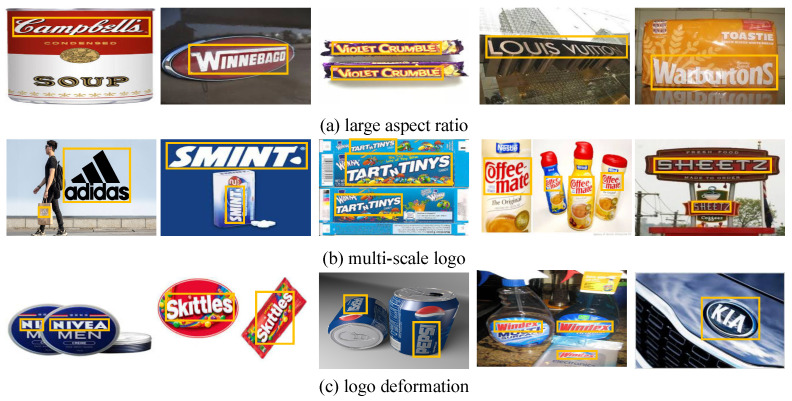
Three logo challenges. (**a**) Logo with a large aspect ratio; (**b**) logos with multiple scales in an image; (**c**) logo deformation caused by angle change, reflection, and other reasons.

Compared with general object detection, the challenges of logo detection mainly come from two aspects:

- The large aspect ratio of logos usually spans a large area in an image, as shown in Figure 1a. As far as we know, there has been little research on the problem of large aspect ratios in logo detection. Existing two-stage approaches based on fixed-size anchors fail to complete the detection of flexible aspect ratio logos [5,6,7,8]. Optimized strategies [9,10,11] also have a limited effect on the detection of logos with a large aspect ratio. The method in [12] could generate anchors of any shape, but it was unable to extract long-range dependence. Similarly, the traditional convolutional approach cannot fully utilize long-range interaction and the locations of various spatial features, which severely restricts its ability to address the large aspect ratio of logos.
- Multiscale logo objects in an image. As seen in Figure 1b, ’adidas’ appears both in the foreground and background, but the scale varies greatly. Scale diversity can be resolved utilizing feature pyramid networks (FPNs) [13], but the semantic information of small objects may be lost after multiple instances of downsampling. The bottom–up information channel is increased by PANet [14], but the information is concentrated more in the adjacent layers. Although SEPC [15] can extract multilevel features, it has the disadvantage of the topology being too simple to extract more information.

In this paper, we present a novel logo detection method called long-range dependence involutional Network (LDI-Net). We rethink the intrinsic principle of convolution, and propose long-range dependence involution (LD involution) and apply it to a region proposal network (RPN). Two major convolutional flaws are remedied by LD involution, since it has significant advantages in acquiring long-range interactions in spatial and channel dimensions. Meanwhile, it can preferentially extract significant visual information in space by creating particular involutional kernels for certain spatial locations. The construction of LD involution enables visual information and elements in the spatial domain to be reasonably allocated and sorted on the logo image to the greatest extent. The channel-sharing involutional kernel allows for us to use a larger K to satisfy the establishment and correlation of long-range information, and significantly reduces the redundancy of the model. For logo detection, a logo image with a very large aspect ratio is characterized by high requirements for long-distance information contact. LDI-Net improves the detection performance of logos with a large aspect ratio by employing a new operator and a self-attention mechanism. For the second issue, we suggest a multilevel representation neural architecture search (MRNAS) to detect multiscale logo objects. MRNAS introduces six heterogeneous information paths to construct a diverse multipath topology that combines semantic information and location representation, optimizing cross-level interaction between features. Additionally, we implemented an adaptable RoI pooling module (ARM) to improve detection efficiency and achieve adaptive feature learning for differently shaped objects. By adding additional offset and a modulation mechanism, the logo deformation problem caused by angles, occlusion, rotation, distortion, reflection, etc. (as shown in Figure 1c) is solved.

The main contributions of this paper can be summarized as follows:

- We developed a network with LD Involution for logo detection by establishing long-range information dependence, and ranking the significance of visual information via a new operator and a self-attention mechanism to solve the problem of a large aspect ratio.
- We constructed a diverse multipath topology on the basis of neural architecture search theory in which each path utilizes a specific feature fusion.
- We conducted extensive experiments and evaluated our approach on four benchmark logo datasets: FlickrLogos-32, QMUL-OpenLogo, LogoDet-3K-1000 and LogoDet-3K. The experimental results demonstrate the effectiveness of the proposed model.

## 2. Related Work

### 2.1. Object Detection

In recent years, CNNs have been widely used in deep learning and have achieved many good research results [16,17,18]. Object detection is one of the most fundamental and challenging problems in computer vision, and has received much attention in recent years. In the era of deep learning, object detection methods are divided into two genres: two-stage and one-stage. The two-stage system first creates regional proposals on the basis of image content, followed by categorization and localization. Classical two-stage algorithms include fast R-CNN [19], faster R-CNN [5], and cascade R-CNN [20]. One-stage algorithms are characterized by one-step completion without regional proposals, directly generating the category and location coordinates, such as the YOLO series [8,21,22]. Among them, faster R-CNN is a milestone work based on RPN.

It is an important issue for object detection to recognize multiscale objects. Many works were improved on the basis of FPN [13], including PANet [14], BiFPN [23], and SEPC [15], because of its strong performance in multilevel feature extraction. PANet enhanced the representation ability by integrating bottom–up and top–down paths. BiFPN introduced learnable weights to determine the importance of different input features, and repeatedly employed multiscale feature fusion. SEPC performed deformable convolution on the high-level features of a feature pyramid, which adapted to the actual scale change and maintained scale balance between layers. Although these methods implemented the information interaction between multiple layers, the relatively simple topology of the search structure lacked the feature information of small objects.

### 2.2. Logo Detection

Logo detection has been extensively studied in e-commerce and multimedia fields [24,25,26,27]. Early logo detection was generally completed on the basis of manual features and traditional classification models, such as Viola–Jones (VJ) [28], the histogram of oriented gradients (HOG) [29], and the deformable parts model (DPM) [30]. Yan et al. [31] used the Bayesian classifier framework to detect and remove video logos. Wang et al. [32] implemented a simple automotive logo recognition method using template matching and edge orientation histograms.

In the last few years, deep-learning-based logo detection algorithms have become mainstream. Bao et al. [33] directly applied faster R-CNN to logo detection and achieved good performance. Xu et al. [27] proposed a solution to robust defence competition in e-commerce logo detection. Velazquez et al. [34] improved the detection performance of small objects by incorporating FPN into the DETR structure. Wang et al. [25] built the largest fully annotated logo detection dataset, i.e., LogoDet-3K, and proposed Logo-Yolo to resolve the imbalanced samples of logo objects. A cross-view learning method [35] provided ideas for logo detection. Hou et al. [26] constructed a large dataset, FoodLogoDet-1500, to address data limitations in food logo detection, and proposed MFDNet to address multiscale and similar logo problems.

Different from previous work, we rethought the intrinsic principle of convolution and applied the proposed LD involution to RPN. Meanwhile, we constructed a diverse multipath topology on the basis of neural architecture search theory in which each path utilized a specific feature fusion. In addition, we introduced ARM to achieve adaptive feature learning for different objects.

## 3. Our Approach

**Figure 2:**
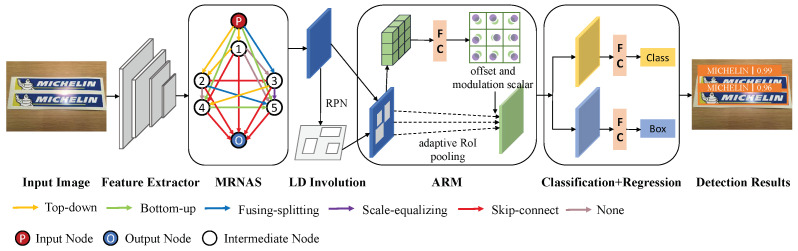
Overview of proposed LDI-Net for logo detection. MRNAS: multilevel representation neural architecture search. LD involution: long-range dependence involution. ARM: adaptive RoI pooling module.

In this section, we present logo detection method LDI-Net, and the overall framework is shown in Figure 2. Specifically, the model first feeds the feature map into MRNAS to learn multilevel features after extracting essential features from the input image. Next, it feeds the feature map into RPN, established by LD involution to obtain higher-quality regional proposals. Then, it feeds the feature map into ARM to enhance the modeling capability. Lastly, the model performs classification and localization. All components are described in detail in the following sections.

### 3.1. Multilevel Representation Neural Architecture Search

As shown in Figure 2, the main body of MRNAS is a fully connected directed acyclic graph composed of *N* + 2 nodes, while *N* is a predefined constant value. In LDI-Net, to balance efficiency and accuracy, we set *N* to 5. The nodes of the directed acyclic graph represent the feature map driven by the feature pyramid, *P* is the input node, *O* is the output node, and $t_{i}\left(i=1,2,\ldots ,N\right)$ is the intermediate node. Different information paths are used as connections between the two nodes. We introduced six kinds of heterogeneous information paths: top–down, bottom–up, fusing–splitting, scale–equalizing, skip–connect, and none [36]. They could realize the aggregated combination of multilevel information on different paths. These information paths PA(i,j) transform $t_{i}$ into $t_{j}$, and each node i∈$\left\{1,2,\ldots ,N\right\}$ aggregates the input of the previous node:

(1) $$t_{j}=\sum_{i<j}PA\left(i,j\right)\left(t_{i}\right)$$

### 3.2. Long-Range Dependence Involution

The purpose of RPN is to generate regional proposals when detecting objects. LD involution is a more efficient way to correlate information compared with convolution, which improves the quality of generated candidate regions better than RPN.

**Figure 3:**
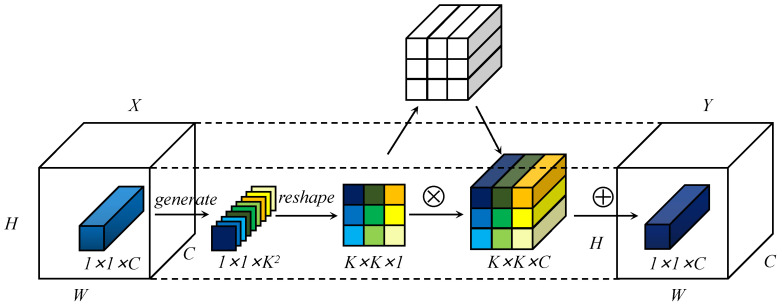
Feature map transformation process based on involution.

**Figure 4:**
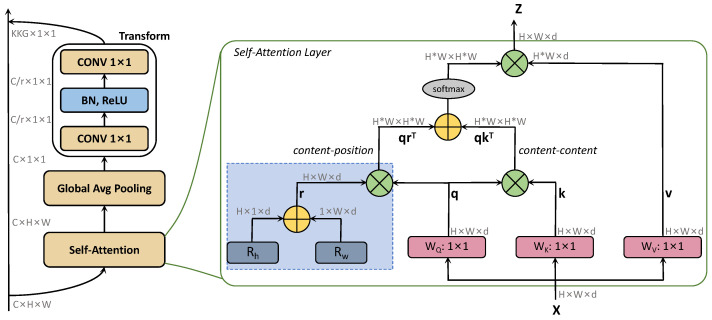
Construction of the involutional kernel.

Similar to involution [37], the feature transformation process of LD involution is shown in Figure 3. For a coordinate point in the input feature map, its feature vector is first transformed by two steps of generation (as given in Figure 4) and each reshaped to expand into the involutional kernel corresponding to the coordinate point. Then, it multiadds with the K×K neighborhood near the coordinate point to obtain the final output feature map.

We focus on long-range dependence, which is crucial for the optimization of large aspect ratios. Inspired by [38], we adopted a flexible generation method to generate the involutional kernel instead of the convolutional kernel. As shown in Figure 4, the involutional kernel was constructed in two parts. In the first part, we used global self-attention to extract distant information. In the content-position section, we utilized relative position encodings $R_{h}$ and $R_{w}$ to represent height and width, respectively. We used *q*, *k*, and *r* to represent query, key, and position encoding, respectively. Attention logits are denoted as $qk^{T}+qr^{T}$. ⨁ and ⨂ represent element-wise and matrix multiplication, respectively. After self-attention, global average pooling was employed to refine the context modeling and enrich the extraction of long-range information. The second part is to capture channel dependence by learning the correlation between channels and filtering attention to the channel. After the feature extraction of different positions in the first part, the channel feature dependence was successively obtained with 1×1 convolution, BN, ReLU, and 1×1 convolution. Combined with the general form described above, the module is defined as follows:

(2) $$M_{i,j}=W_{s}\delta W_{f}\left(G\left(S\left(X_{i,j}\right)\right)\right)$$

where $X_{i,j}$ and $M_{i,j}$ represent input and output, respectively. *S* represents the global self-attention, while *G* represents global average pooling. $W_{s}$ and $W_{f}$ represent the linear transformation matrix (1×1 convolution was adopted here), while δ represents BN and ReLU.

### 3.3. Adaptive RoI Pooling Module

RoI pooling is used to pool arbitrary-size input feature maps into the same size feature maps. RoI pooling divides RoI into *i* bins. Each bin can be formulated as follows:

(3) $$y\left(i\right)=\sum_{t=1}^{m_{i}}x\left(R_{it}\right)/m_{i}$$

where *x* is the input feature map, and *y* is the output feature map. $R_{it}$ is the sampling position of the t−th grid cell in the i−th bin, and $m_{i}$ is the number of grid cells in the bin. We summed the sampling values on the grid cell and took the average value to calculate the output of the bin.

As shown in Figure 2, in the adaptive RoI pooling, we added an additional offset and a modulation mechanism [39]:

(4) $$y\left(i\right)=\sum_{t=1}^{m_{i}}x\left(R_{it}+\Delta R_{i}\right)\cdot \Delta h_{i}/m_{i}$$

where $\Delta R_{i}$ is the offset that is used to increase the spatial sampling position and improve the feature extraction ability of the network. $\Delta h_{i}$ is the modulation scalar that is used to assign the weight to each offset corrected region.

### 3.4. Loss Function

In LDI-Net, the final loss function consists of $L_{rpn}$, $L_{cls}$ and $L_{loc}$, as listed in Equation (5):

(5) $$L=L_{rpn}+L_{cls}+L_{loc}$$

where $L_{rpn}$ is the RPN loss, $L_{cls}$ is the classification loss, and $L_{loc}$ is the boundary box regression loss.

We implemented $L_{cls}$ by the cross-entropy loss function. In order to better adapt the changes in distribution, we used Dynamic SmoothL1 Loss (DSL) in $L_{loc}$ to compensate for high-quality samples and pay more attention to high-quality samples:

(6) $$DSL\left(a,\sigma \right)=\left\{\begin{array}{cc} {0.5|a|}^{2}/\sigma , & {if|a|}^{2}<\sigma ,\\ |a|-0.5\sigma , & otherwise. \end{array}\right.$$

## 4. Experiments

### 4.1. Experimental Setting

#### 4.1.1. Datasets

To evaluate the effectiveness of the proposed LDI-Net, we completed comprehensive experimental validation on four datasets: large-scale dataset LogoDet-3K [25], medium-scale dataset LogoDet-3K-1000 [25], and two small-scale datasets, QMUL-OpenLogo [40] and FlickrLogos-32 [41]. LogoDet-3K contains 158,652 pictures, including 142,142 for trainval and 16,510 for the test. LogoDet-3K-1000 is a subset of LogoDet-3K, sampled from LogoDet-3K. To further evaluate the generalization and robustness of the LDI-Net model, we also carried out extensive experiments on two widely used logo detection datasets, i.e., QMUL-OpenLogo and FlickrLogos-32. The detailed description of these datasets is shown in Table 1. The classes, images and objects represent the number of categories, images and logos in the dataset, respectively. The trainval and test represent a division of the dataset whose sum is the number of images.

#### 4.1.2. Implementation Details

We implemented our method on the basis of the publicly available MMDetection toolbox [42], and used dynamic R-CNN [43] based on ResNet-50 as the baseline. We chose ResNet-50 as the backbone network because of its two advantages: (1) ResNet-50 itself had little influence on the model, which rendered the improvement effect of the proposed model more obvious. (2) It is beneficial for researchers to conduct comparisons in the experiments since it is a classical network that has been widely used. For evaluation, we used the widely used mean average precision (mAP) [44] with an IoU threshold of 0.5. Meanwhile, we added processing time, model size, parameters, and FLOPs in order to further detail the experimental results. Processing time refers to the time from the beginning of the training process to convergence. The model size, parameters, and FLOPs can provide a reference for measuring the model complexity. In our experiments, the basic detection network was trained using stochastic gradient descent (SGD), and the initial learning rate was set to 0.002. In the data preprocessing stage, all input images were resized into 1000 × 600. The weight decay was 0.0001, and the momentum was 0.9. We followed the settings in MMDetection for the other hyperparameters.

### 4.2. Experiments on LogoDet-3K

#### 4.2.1. Comparisons with State of the Art

We compared the proposed LDI-Net with several other one-stage and two-stage popular baselines, as reported in Table 2.

Table 2 shows the best detection performance of all methods with a uniform learning rate. The proposed LDI-Net method was superior to other baselines, as it achieved the best performance with 88.7% mAP. It achieved 4.9% and 1.6% improvements over faster R-CNN and dynamic R-CNN, respectively. The proposed LDI-Net strategy also achieved the best performance compared to other approaches that utilize feature fusion. For example, PANet, Libra R-CNN, and our method all utilize feature fusion to extract multilevel features. In comparison, LDI-Net achieved 5.6% and 5.3% improvement over PANet and Libra R-CNN, respectively. To verify the detection of large aspect ratio logos, we also compared guided anchoring and achieved 2.4% accuracy improvement, which also shows our method’s advantages in long-range interactions. In comparison with the other baselines, the proposed LDI-Net improved mAP by 10.4%, 8.8%, 7.5%, 6.6%, 4.3%, 5.2%, 6.0% 2.0% and 14.4% compared with FSAF, ATSS, GFL, Soft-NMS, generalized IoU, distance IoU, complete IoU, SABL and sparse R-CNN, respectively.

#### 4.2.2. Qualitative Analysis

**Figure 5:**
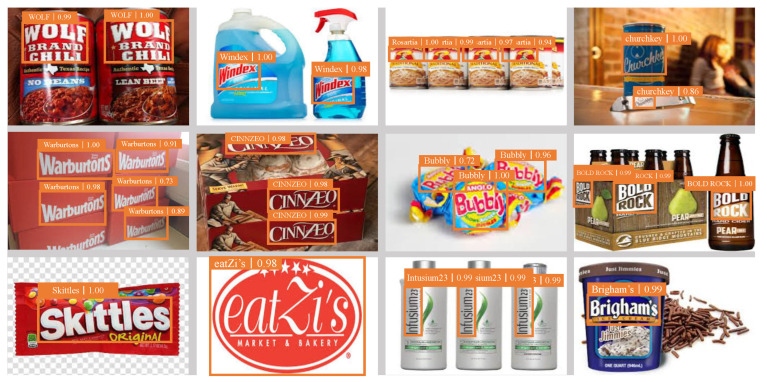
Some examples of LDI-Net test results. The orange box represents the location of the detected logo object. The top of the box represents categories and accuracy.

In Figure 5, we present some illustrative examples for LDI-Net. Our model could achieve good detection performance on regular large logos and logos with large aspect ratios. For example, the detection of Warburtons and CINNZEO showed good performance on multiscale logos, while the detection of Bubbly and BOLD ROCK showed great results on deformed logos in the second line. Our model also achieved over 98% detection accuracy on Skittles and eatZis with a large proportion, and logos with disparate aspect ratios (e.g., Intusium23 and Brigham’s) in the third line.

**Figure 6:**
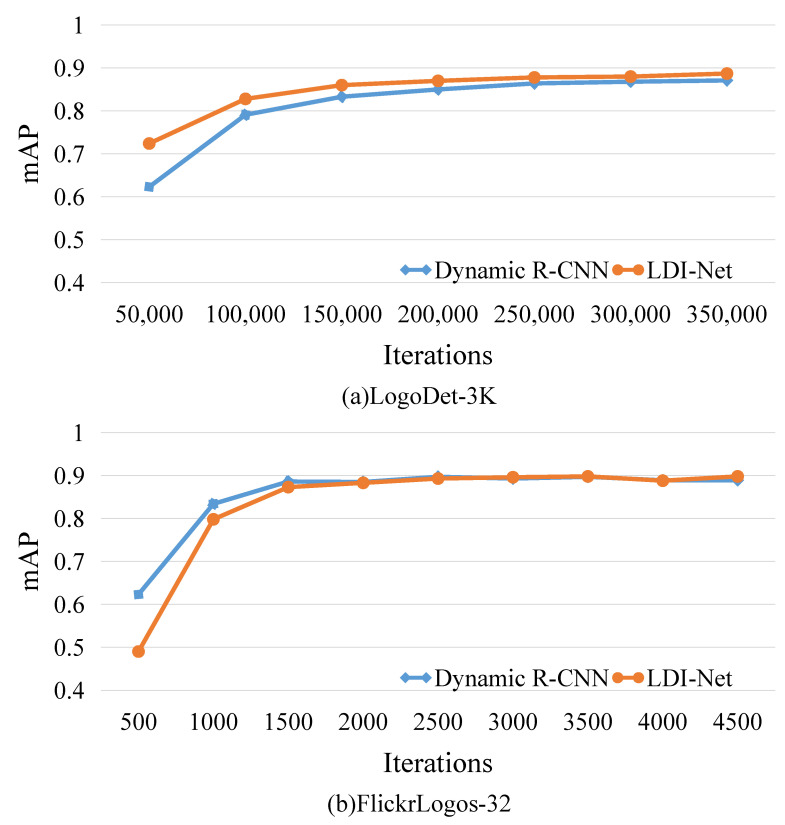
Comparison of dynamic R-CNN and LDI-Net with increasing number of iterations.

We also set up different iterations to compare the proposed strategy and dynamic R-CNN in terms of convergence and accuracy. Figure 6 provides the performance trend when the iterations increased, showing that our method gradually stabilized, starting from 250,000 and converging at 350,000. During the training process, it was clear that our method maintained a higher mAP than that of dynamic R-CNN.

### 4.3. Experiments on Other Benchmarks

#### 4.3.1. Results on LogoDet-3K-1000

LogoDet-3K-1000 is a subdataset of LogoDet-3K that has a suitable number of images and categories. Experiments on this dataset helped in further evaluating our model. We used different strategies on LogoDet-3K-1000 and list the results in Table 3. The proposed strategy outperformed other baseline approaches and achieved 90.4% mAP. In detail, it achieved 1.3%, 2.0%, 1.3%, 1.9%, and 3.6% improvement compared with PANet, Libra R-CNN, guided anchoring, dynamic R-CNN, and sparse R-CNN, respectively.

#### 4.3.2. Results on QMUL-OpenLogo

We provide experimental results on QMUL-OpenLogo to verify the effectiveness of LDI-Net. As shown in Table 4, our model obtained 56.3% mAP, which outperformed all the other baselines. It achieved 2.5% and 14.7% improvements compared with classical algorithms faster R-CNN and SSD, respectively. Both dynamic R-CNN and Libra R-CNN achieved 54.6% mAP, our method still achieved a 1.7% improvement. Our method was also superior to feature fusion-based methods, e.g., PANet. These comparisons further verify the superiority of our method in information exchange and feature fusion.

#### 4.3.3. Results on FlickrLogos-32

We also performed a comparison on FlickrLogos-32. Table 5 shows that our algorithm achieved a significant improvement compared to the base algorithms, and had the best performance with 89.8% mAP. For example, it achieved 2.7% and 1.6% mAP improvement compared with one-stage algorithm GFL and two-stage algorithm faster R-CNN, respectively. Guided anchoring is an improvement on anchor, and our method outperformed it by 1.4%. These results indicate that the proposed method is efficient in detecting logos with a large aspect ratio. Dynamic R-CNN and LDI-Net showed similar performance trends on FlickrLogos-32, as shown in Figure 6b, because of the poor quantity and quality of images in the dataset.

### 4.4. Ablation Study

In this section, we conduct comprehensive analysis of the effects of each LDI-Net component on four logo datasets. We compare the test and localization accuracy of each LDI-Net component with dynamic R-CNN, namely, LD involution, MRNAS, and ARM. We used dynamic R-CNN equipped with ResNet-50 and FPN as the baseline.

#### 4.4.1. LD Involution

LD involution is a targeted solution to the large aspect ratio problem. As shown in Table 6, LD involution achieved 87.3% mAP, outperforming other baselines on LogoDet-3K. Table 7 shows that our method outperformed the baseline by 1.2% improvement on LogoDet-3K-1000. Our method also achieved 1.5% and 0.7% improvement on QMUL-OpenLogo and FlickrLogos-32, as shown in Table 8 and Table 9, respectively. In the comparison with involution, our method also showed superiority. In particular, on the QMUL-OpenLogo, our method achieved 1% improvement.

**Figure 7:**
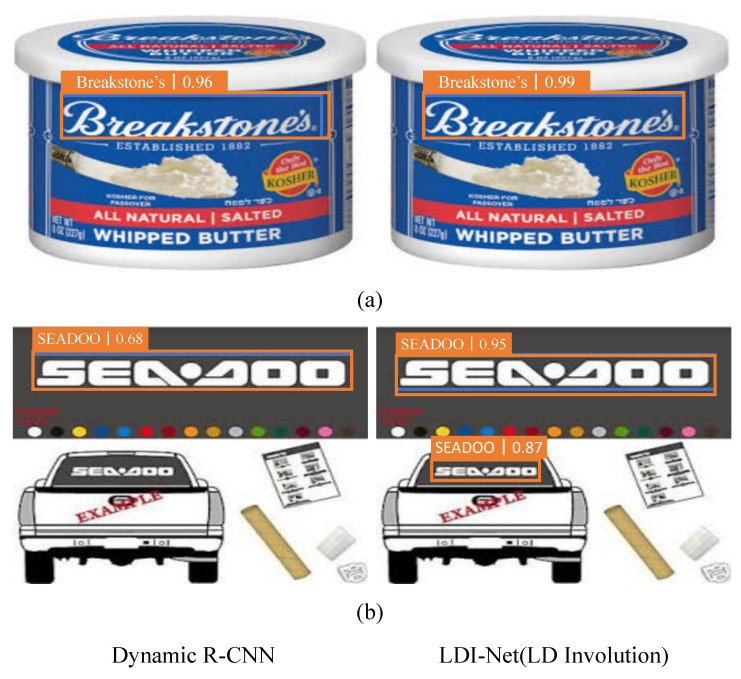
Comparison of visualization results of dynamic R-CNN and LDI-Net for the large aspect ratio problem. Blue boxes: ground-truth boxes. Orange boxes: correct detection boxes.

Figure 7 shows the visualization comparison results of dynamic R-CNN and LD involution on LogoDet-3K. A logo image with a large aspect ratio is taken as the visual displaying result, which shows that the accuracy of our method was higher than the baseline. For example, for the logo with a very wide aspect ratio, our model achieved 3% and 27% improvement over the baseline, as shown in Figure 7a,b, respectively. In addition, LD involution could identify small logo ‘SEADOO’, while the baseline could not, as shown in Figure 7b. This indicates that LD involution extracting long-range information is also effective for small objects with a large aspect ratio.

#### 4.4.2. MRNAS

We applied the MRNAS module to solve multiscale problems and achieved good results. The MRNAS module performed well on two large logo detection datasets, i.e., LogoDet-3K and LogoDet-3K-1000. As shown in Table 6, on LogoDet-3K, the mAP of our model with MRNAS reached 87.5%, a 0.4% improvement over dynamic R-CNN. On LogoDet-3K-1000, the model with MRNAS reached 89.5% mAP, achieving 1% improvement over the baseline, as seen in Table 7. In addition, MRNAS achieved good performance on the other two datasets, in which mAP was significantly improved, as can be seen in Table 8 and Table 9.

**Figure 8:**
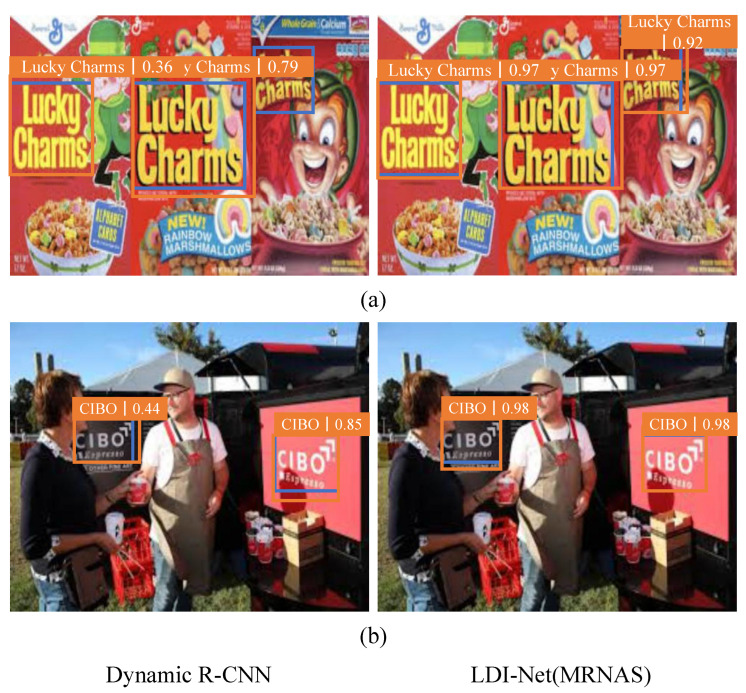
Comparison of visualization results of dynamic R-CNN and LDI-Net for multiscale logo images. Blue boxes: ground-truth boxes. Orange boxes: correct detection boxes.

We provide some illustrative examples of logos with different scales from LogoDet-3K, as shown in Figure 8. In the first pair, the baseline could not detect the rightmost logo. In contrast, our method had a detection accuracy of 92%. Meanwhile, LDI-Net improved the detection mAP from 44% to 98% compared with dynamic R-CNN, which also shows the superiority of the proposed method in the second pair.

#### 4.4.3. ARM

We conducted ablation experiments for ARM on four datasets, and the experimental results demonstrate that ARM works better than the baselines. As shown in Table 6, the performance of a single ARM module was comparable to that of two other modules, up to 88.2% on LogoDet-3K. In the other three datasets (Table 7, Table 8 and Table 9), ARM also achieved an improvement in mAP. The results indicate that ARM can be an effective solution to logo deformation.

**Figure 9:**
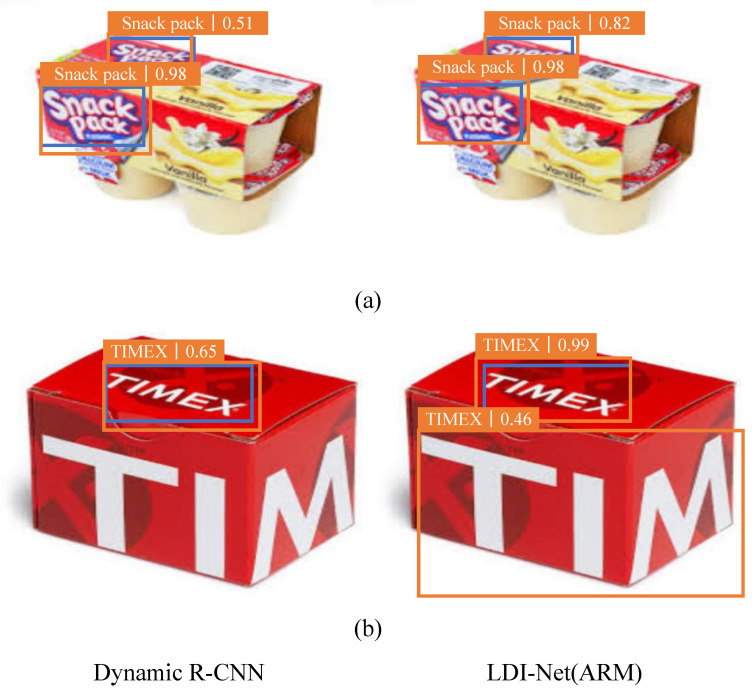
Comparison of visualization results of dynamic R-CNN and LDI-Net for logo deformation images. Blue boxes: ground-truth boxes. Orange boxes: correct detection boxes.

We selected a variety of deformed logos for different reasons to fully illustrate the functionality of ARM in a visualization experiment. Figure 9a shows that our model could still achieve 82% detection accuracy on the logo that was deformed due to the camera angle. As shown in Figure 9b, our model could detect incomplete logo ‘TIMEX’, which confirms the effectiveness of our model.

After testing the components individually, we conducted experiments combining LD involution and MRNAS to further validate the model effects, as shown in Table 6, Table 7, Table 8 and Table 9. On all four datasets, the combination of LD involution and MRNAS performed better than adding only one module.

## 5. Conclusions and Future Work

In this paper, we proposed a logo detection model, long-range dependence involutional network (LDI-Net), to detect logos with large aspect ratios by adding a new operator and a self-attention mechanism. Meanwhile, MRNAS was proposed to construct a novel multipath topology to realize multiscale logo detection. ARM was also introduced to enhance the ability of the proposed model to handle logo deformation.

So far, LDI-Net has worked well, but there are some limitations. Although multiscale logos can be completed well, there is still room for further improvement in the localization and classification of some small logos. Our method could also solve the problem of logo deformation caused by occlusion and rotation very well, but the deformations caused by reflection and distortion need to be studied more specifically. In future work, we will continue to conduct indepth research to solve the above problems. Further, we will address other challenges of logo detection, such as small, similar, and low-resolution logos.

## Figures and Tables

**Table 1:** Statistics of four logo datasets.

Datasets	#Classes	#Images	#Objects	#Trainval	#Test
FlickrLogos-32 [41]	32	2240	3405	1478	762
QMUL-OpenLogo [40]	352	27,083	51,207	18,752	8331
LogoDet-3K-1000 [25]	1000	85,344	101,345	75,785	9559
LogoDet-3K [25]	3000	158,652	194,261	142,142	16,510

**Table 2:** Detection results on LogoDet-3K.

Methods	Backbone	mAP(%)	Processing Time (days)	Size (KB/epoch)	Params (M)	FLOPs (G)
One-stage:						
FSAF [45]	ResNet-50-FPN	78.3	5	336,668	42.92	349.84
ATSS [46]	ResNet-50-FPN	79.9	7	304,457	38.8	348.86
GFL [47]	ResNet-50-FPN	81.2	5	305,590	38.95	**351.96**
Two-stage:						
Faster R-CNN [5]	ResNet-50-FPN	83.8	4	442,663	56.49	222.02
Soft-NMS [48]	ResNet-50-FPN	82.1	-	-	56.28	177.46
PANet [14]	ResNet-50-PAFPN	83.1	5	470,332	60.03	246.8
Cascade R-CNN [20]	ResNet-50-FPN	85.6	8	611,864	78.15	243.68
Generalized IoU [49]	ResNet-50-FPN	84.4	7	442,663	56.49	222.02
Libra R-CNN [50]	ResNet-50-BFP	82.4	5	444,726	56.76	223.07
Guided Anchoring [12]	ResNet-50-FPN	86.3	-	-	57.08	221.79
Distance IoU [51]	ResNet-50-FPN	83.5	4	442,663	56.49	222.02
Complete IoU [51]	ResNet-50-FPN	82.7	4	442,663	56.49	222.02
Dynamic R-CNN [43]	ResNet-50-FPN	87.1	8	442,664	56.49	222.02
SABL [52]	ResNet-50-FPN	85.7	6	352,738	44.98	269.34
Sparse R-CNN [53]	ResNet-50-FPN	74.3	5	1,297,338	110.57	150.36
**LDI-Net(ours)**	**ResNet-50-MRNAS**	**88.7**	**10**	**1,443,658**	**183.66**	152.1

**Table 3:** Detection results on LogoDet-3K-1000.

Methods	Backbone	mAP(%)
One-stage:		
FSAF [45]	ResNet-50-FPN	87.3
ATSS [46]	ResNet-50-FPN	87.8
GFL [47]	ResNet-50-FPN	87.7
Two-stage:		
Faster R-CNN [5]	ResNet-50-FPN	88.2
Soft-NMS [48]	ResNet-50-FPN	89.1
PANet [14]	ResNet-50-PAFPN	89.1
Cascade R-CNN [20]	ResNet-50-FPN	89.1
Generalized IoU [49]	ResNet-50-FPN	88.2
Libra R-CNN [50]	ResNet-50-BFP	88.4
Guided anchoring [12]	ResNet-50-FPN	89.1
Distance IoU [51]	ResNet-50-FPN	88.7
Complete IoU [51]	ResNet-50-FPN	88.9
Dynamic R-CNN [43]	ResNet-50-FPN	88.5
SABL [52]	ResNet-50-FPN	88.8
Sparse R-CNN [53]	ResNet-50-FPN	86.8
**LDI-Net(ours)**	**ResNet-50-MRNAS**	**90.4**

**Table 4:** Detection results on QMUL-OpenLogo.

Methods	Backbone	mAP(%)
One-stage:		
SSD [6]	VGG-16	41.6
FSAF [45]	ResNet-50-FPN	44.6
ATSS [46]	ResNet-50-FPN	48.4
GFL [47]	ResNet-50-FPN	47.3
FoveaBox [54]	ResNet-50-FPN	35.6
Two-stage:		
Faster R-CNN [5]	ResNet-50-FPN	53.8
Soft-NMS [48]	ResNet-50-FPN	54.1
PANet [14]	ResNet-50-PAFPN	54.5
Cascade R-CNN [20]	ResNet-50-FPN	54.2
Generalized IoU [49]	ResNet-50-FPN	54.2
Libra R-CNN [50]	ResNet-50-BFP	54.6
Guided Anchoring [12]	ResNet-50-FPN	52.2
Distance IoU [51]	ResNet-50-FPN	54.4
Complete IoU [51]	ResNet-50-FPN	53.7
Dynamic R-CNN [43]	ResNet-50-FPN	54.6
Double-head R-CNN [55]	ResNet-50-FPN	54.2
SABL [52]	ResNet-50-FPN	53.4
Sparse R-CNN [53]	ResNet-50-FPN	50.5
**LDI-Net(ours)**	**ResNet-50-MRNAS**	**56.3**

**Table 5:** Detection results on FlickrLogos-32.

Methods	Backbone	mAP(%)
One-stage:		
SSD [6]	VGG-16	80.2
RetinaNet [7]	ResNet-50-FPN	78.4
FSAF [45]	ResNet-50-FPN	86.3
ATSS [46]	ResNet-50-FPN	86.4
GFL [47]	ResNet-50-FPN	87.2
FoveaBox [54]	ResNet-50-FPN	85.5
Two-stage:		
Deep Logo [56]	VGG-16	74.4
Faster R-CNN [5]	ResNet-50-FPN	88.2
BD-FRCN-M [57]	VGG-16	73.5
Soft-NMS [48]	ResNet-50-FPN	88.8
PANet [14]	ResNet-50-PAFPN	89.2
Cascade R-CNN [20]	ResNet-50-FPN	89.2
Generalized IoU [49]	ResNet-50-FPN	88.7
Libra R-CNN [50]	ResNet-50-BFP	89.5
Guided Anchoring [12]	ResNet-50-FPN	88.5
Distance IoU [51]	ResNet-50-FPN	88.7
Complete IoU [51]	ResNet-50-FPN	89.0
Dynamic R-CNN [43]	ResNet-50-FPN	88.9
Double-head R-CNN [55]	ResNet-50-FPN	89.2
SABL [52]	ResNet-50-FPN	88.4
Sparse R-CNN [53]	ResNet-50-FPN	81.6
**LDI-Net(ours)**	**ResNet-50-MRNAS**	**89.8**

**Table 6:** Evaluating individual components on LogoDet-3K.

Involution	LD Involution	MRNAS	ARM	mAP(%)
				87.1
✓				87.2
	✓			87.3
		✓		87.5
			✓	88.2
	✓	✓		88.2
	✓	✓	✓	**88.7**

**Table 7:** Evaluating individual components on LogoDet-3K-1000.

Involution	LD Involution	MRNAS	ARM	mAP(%)
				88.5
✓				89.6
	✓			89.7
		✓		89.5
			✓	88.9
	✓	✓		89.9
	✓	✓	✓	**90.4**

**Table 8:** Evaluating individual components on QMUL-OpenLogo.

Involution	LD Involution	MRNAS	ARM	mAP(%)
				54.6
✓				55.1
	✓			56.1
		✓		56.0
			✓	54.8
	✓	✓		56.2
	✓	✓	✓	**56.3**

**Table 9:** Evaluating individual components on FlickrLogos-32.

Involution	LD Involution	MRNAS	ARM	mAP(%)
				88.9
✓				89.2
	✓			89.6
		✓		89.7
			✓	89.2
	✓	✓		89.8
	✓	✓	✓	**89.8**

## Data Availability

The data are contained within the article.
